# Non-Small-Cell Lung Cancer Patients with Coexistence of High PD-L1 Expression and *RET* Fusion—Which Path Should We Follow? Case Reports and Literature Review

**DOI:** 10.3390/jcm11061630

**Published:** 2022-03-15

**Authors:** Magdalena Knetki-Wróblewska, Kamila Wojas-Krawczyk, Dariusz M. Kowalski, Maciej Krzakowski

**Affiliations:** 1 Department of Lung Cancer and Chest Tumours, Maria Skłodowska-Curie National Research Institute of Oncology, 02-781 Warsaw, Poland; dariusz.kowalski@pib-nio.pl (D.M.K.); maciej.krzakowski@pib-nio.pl (M.K.); 2 Department of Pneumonology, Oncology and Allergology, Medical University of Lublin, 20-090 Lublin, Poland; kamilawojas@wp.pl

**Keywords:** immunotherapy, non-small-cell lung cancer, *RET*-fusion, rare molecular alterations, case report

## Abstract

Pembrolizumab is widely used in first-line treatment in patients with advanced non-small-cell lung cancer (NSCLC) with high PD-L1 expression. The activity of pembrolizumab in NSCLC patients with rare molecular alterations is poorly characterised. *RET* gene rearrangements are identified in 1–2% of lung cancer patients. Here, we present two cases of *RET*-rearranged NSCLC patients with high PD-L1 expression (>50%), treated with pembrolizumab within routine clinical practice. Pembrolizumab was ineffective in both cases—single-agent immunotherapy seems to be of limited value in this group of patients. Selective RET-inhibitors, if available, are the optimal treatment for patients with *RET* fusion nowadays. The best sequence of the therapy is still not defined.

## 1. Introduction

Immunotherapy (as monotherapy or combined with chemotherapy) is the accepted standard of care in first-line treatment of advanced non-small cell lung cancer (NSCLC). Pembrolizumab monotherapy in patients with advanced and previously untreatedNSCLC patients with programmed-cell death ligand-1 (PD-L1) tumour proportion score (TPS) of 50% or greater offers significant benefit over chemotherapy [1]. Updated data from the KEYNOTE-024 study show that median overall survival (mOS) was 26.3 months (95% CI, 18.3–40.4) for pembrolizumab and 13.4 months (9.4–18.3) for chemotherapy (HR, hazard ratio, 0.62; 95% CI, 0.48–0.81). The percentage of patients remaining in follow-up after 5 years was 31.9% and 16.3%, respectively [2].

The data on immunotherapy efficacy in patients with molecular abnormalities are based on the retrospective analysis of a small subgroup of patients and the level of evidence for the single anti-PD-1 or anti-PD-L1 agents’ efficacy is very low. Consequently, the decision to include these patients in immunotherapy requires a very careful approach [3,4,5].

The rearranged during transfection gene (*RET* gene), is a proto-oncogene which can be activated via point-mutation or rearrangement [6]. RET gene fusions have been reported in 1–2% of NSCLC patients; the most frequent fusion partner is *KIF5B*. Patients with *RET* gene fusions usually are younger, have non-squamous NSCLC and are non-smokers [7]. Platinum-based and pemetrexed chemotherapy remains the standard of care, with an objective response rate of about 50% and median progression free survival (mPFS) of 9–18 months [8,9]. Non-selective multikinase inhibitors, such as lenvatinib, vandetanib and cabozantinib, have also been evaluated prospectively. In the case of those drugs, the objective response rate ranged from 16% to 47% and mPFS from 4.7 to 7.3 months [10,11,12]. The results from the ARROW and LIBRETTO-001 studies evaluating pralsetinib and selpercatinib, selective RET inhibitors, have been recently published [7,13,14]. Both agents produced objective response rate (ORR) of over 60%. For 39 patients who received selpercatinib as first-line treatment ORR was 85% and in 90% of patients the duration of response (DOR) exceeded 6 months. High intracranial activity of pralsetinib and selpercatinib should also be noted [7,13,14].

Here, we described two patients with *RET*-positive advanced NSCLC and PD-L1 expression greater than 50% who were treated with pembrolizumab in the first-line setting. Additionally, we had analysed the available data in the current literature. The data indicate that the place for immunotherapy in this group of patients and in patients with other rare molecular alterations is still disputable.

## 2. Case 1

**Figure 1:**
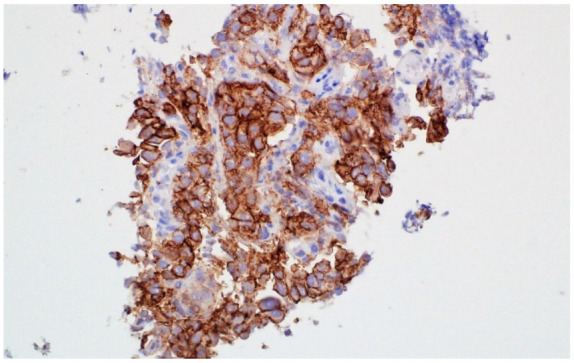
Immunohistochemistry analysis of PD-L1 expression on tumour cells using 22C3 anti-PD-L1 antibody (DAKO) (PD-L1 TPS—90%).

A 69-year-old man, never-smoker, without significant comorbidities was diagnosed in March 2020 due to a cough. Pulmonary adenocarcinoma was diagnosed based on bronchial specimen. Immunohistochemistry analysis showed 90% of PD-L1 TPS (staining was performed with 22C3 antibody clone; Figure 1).

**Figure 2:**
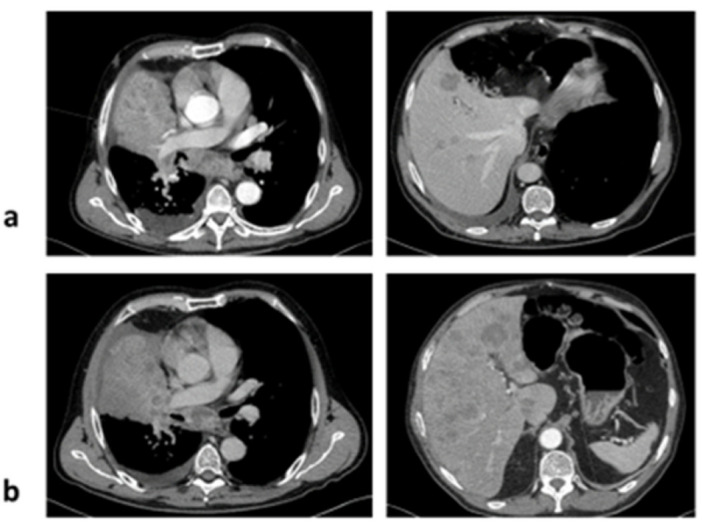
Computer tomography scans: (**a**) at baseline, and (**b**) at disease progression after two cycles of pembrolizumab.

Molecular testing was performed with the next generation sequencing (NGS) technique using FusionPlex Comprehensive Thyroid and Lung (CTL) Kit and sequenced on MiniSeq (Illumina). *KIF5B-RET* fusion was confirmed. Staging procedures showed stage IV disease (lung, bones and liver metastases). The patient was in a good general condition, ECOG 1, and started pembrolizumab (200 mg i.v. every 21 days) in April 2020. After two doses, he experienced severe back pain—a pathological fracture of Th10 was diagnosed. Computed tomography (CT) revealed progressive disease (PD) in form of new liver metastases and increase of lung lesions (Figure 2). The subsequent systemic therapy was not administered. Palliative chest radiotherapy was initiated, but patient general condition deteriorated rapidly, and he died in July 2020 (3 months after treatment initiation).

## 3. Case 2

**Figure 3:**
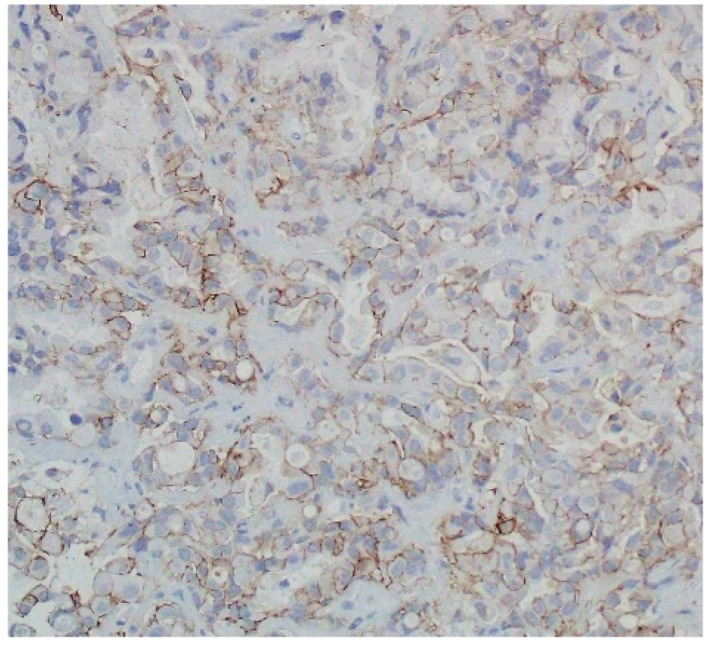
Immunohistochemistry analysis of PD-L1 expression on tumour cells using 22C3 anti-PD-L1 antibody (DAKO) (PD-L1 TPS—70%).

A 65-year-old woman, in a good general condition, ECOG 1, never-smoker, without serious comorbidities was diagnosed in December 2019 due to chest pain and cough. The diagnosis of adenocarcinoma with 70% of PD-L1 TPS was established based on liver biopsy specimen investigation (immunohistochemical staining was performed with 22C3 antibody clone; Figure 3).

**Figure 4:**
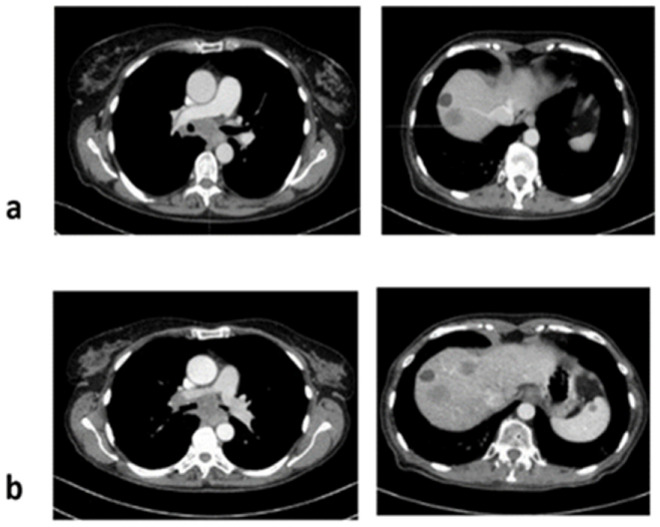
Computer tomography scans: (**a**) at baseline, and (**b**) at disease progression after four cycles of pembrolizumab.

Molecular testing confirmed *CCDC6-RET* fusion. Stage IV disease was diagnosed (lung, bones, and liver metastases) and pembrolizumab treatment was initiated in February 2020. Progressive disease (mediastinal lymph nodes progression, new lung nodules, bone lesions, and liver metastases) was found on CT which was performed three months later in May 2020—the patient received four doses of pembrolizumab (200 mg i.v. every 21 days) (Figure 4). Then, palliative chest radiotherapy was performed. Afterwards, due to the poor performance status, she was not qualified for chemotherapy. Best supportive care (BSC) was offered. The patient died in November 2020 (nine months after diagnosis).

## 4. Discussion

*RET* gene rearrangements are identified in 1–2% of patients with NSCLC and in some of them, high PD-L1 expression coexists (13–50% of patients in small published cohorts) [7,15,16]. High PD-L1 expression is an established positive predictive factor for immunotherapy in the general population. In the presented case reports, two NSCLC patients with *RET* gene fusion did not achieve any clinical benefit after first line pembrolizumab-based therapy despite the high expression of PD-L1 on neoplastic cells (>50%).

The value of immunotherapy, such as anti-PD-1/PD-L1 monotherapy in patients with molecular alterations, is questionable. It is considered to be of lower efficacy, especially in patients with *EGFR* activating mutations and *ALK* rearrangements [4,16,17,18]. There are several theories that could explain the low activity of this type of treatment.

First of all, single driver mutations in lung cancer are enriched in patients who are never smokers or have low smoking history [4,19]. Moreover, in such cases, lung adenocarcinoma expressed usually lower tumour mutational burden (TMB) than in smokers. A higher number of somatic mutations causes an increased number of neoantigens, which translates into increased immunogenicity of such tissues. Cancers with high TMB are thought to be more sensitive to ICI [4,17]. The lower effectiveness of immunotherapy in non-smoking NSCLC patients who develop only single genetic abnormality, such as *EGFR* mutations or *ALK* rearrangements is well explained [4,18,19].

Secondly, tumour microenvironment in lung cancer with single driver mutation is considered to be “cold, non-inflamed” and that the tumours are scanted of any immune cell infiltration or inflammatory signs. The lower efficacy of immunotherapy in patients with low TMB and low concentration CD8+ tumour-infiltrating lymphocytes (TILs) than in patients with “inflamed” tumours seems to be well documented in the literature [4,17].

### 4.1. The Value of Immunotherapy in Patients with RET Fusion

Several works have been published on the effectiveness of immunotherapy in patients with various rare molecular alterations. The analysed populations included patients with *RET* fusion. Guisier et al. had determined immune checkpoint inhibitors (ICIs) effectiveness in NSCLC patients harbouring *BRAF*, *HER2*, *MET*, and *RET* genes abnormalities in a real-world setting. Among 107 patients, only nine had *RET* translocation. Before ICIs, the patients had received one treatment line. For *RET*-altered patients, mPFS was 7.6 months, median DOR was 4.7 months and the ORR—38% (3 patients—partial response, 2—stable disease, 3—progressive disease, and 1—not evaluable). However, the group of *RET*-altered patients was too few in number to draw reliable conclusions about the effectiveness of ICIs in these patients [19]. Offin et al. had reported ICIs effectiveness in 13 patients with *RET*-rearranged lung cancer with low PD-L1 expression and low TMB. No response was achieved for patients receiving either single anti–PD-L1 or combination with anti-CTLA-4. Offin et al. had no representation of PD-L1-high tumours; therefore, the results are particularly related to a very small subgroup of patients [15]. On the other hand, Hegde et al. showed that in patients with *RET*-positive malignancies (medullary thyroid cancer, NSCLC and other types), time to progression was shorter when treated with ICIs compared to non-ICI therapy [20]. The clinical effectiveness of therapy was related to the type of *RET* pathway aberration: significantly higher risk of progression was observed for *RET*-mutated (not translocated) malignancies treated with ICIs compared to non-ICI therapy [20]. Lee et al. presented the clinical characteristics and the value of various methods of systemic treatment in a group of 59 patients with *RET* fusion (most often KIF5B 21–65.6% and CCDC6 6–18.8%) [21]. Selective RET inhibitors (pralsetinib/selpercatinib) were not used in this group treatment. The best outcomes were achieved using platinum-based and pemetrexed chemotherapy—mPFS of 9.0 months (95% CI: 6.9–11.2) and mOS of 24.1 months (95% CI: 15.2–33.0). Immunotherapy was used in 22% of the patients as first- or second-line treatment (nivolumab, pembrolizumab, atezolizumab), and in two patients combined treatment was administered as third-line treatment (durvalumab/tremelimumab). No objective response was observed in patients receiving PD-1/PD-L1 inhibitors, with disease control rate of 25-50%. Generally, mPFS was 2.1 months (95% CI: 1.6–2.6) and mOS—12.4 months (95% CI: 2.9–21.8) [21] in all patients treated with immunotherapy whose cases were presented in the study. The high percentage of patients with brain metastases during follow-up should be noted; according to the study the metastases were observed in 60% of the patients in 24 months. Low intracranial activity of available treatment methods, including immunotherapy, poses a significant challenge in treatment of patients with *RET* gene fusion. Bhandari et al. have recently published results of analysis using data from Flatiron Health-Foundation Medicine Clinico-Genomic Database and Guardant Health Database [22]. In total, 264 patients with *RET* fusion were identified and 69 of them had received immunotherapy as first- or second-line treatment. Median PFS in patients receiving immunotherapy as first-line treatment was 4.2 months (95%CI 1.4–8.4), while mOS—19.1 months (available data for 17 patients included in Clinico-Genomics Database) [22]. For 12 patients who received chemoimmunotherapy as first-line treatment mPFS was 5.4 months and mOS—19.1 months (6.9-NR); ORR of 70% was noted. The authors confirmed the clinical profile of *RET* positive NSCLC patients, which was mentioned above. The patients were younger, had fewer comorbidities, and lower smoking history than general population of NSCLC patients. TMB and PD-L1 expression data were also presented in the study. They were not complete, as they were available only for 10% of the patients, but PD-L1 expression <1% and TMB < 6 mutations per megabase were observed in most of the cases [22].

The results of the above-mentioned retrospective studies and published case reports evaluating immunotherapy (monotherapy) in *RET*-rearranged patients are summarised in Table 1.

On the other hand, the place of RET inhibitors, with particular focus on the optimal sequence of treatment, is also an important issue. Most patients in the registration studies of selpercatinib and pralsetinib received drugs after the failure of previous therapies. Accordingly, registration records allow for the use of the drugs in previously treated patients. However, it should be noted, that some differences in ORR and mPFS were found depending on prior treatment status. For selpercatinib, ORR was 64% (95% CI: 54–73%) in pre-treated patients, while 85% in untreated patients (95% CI: 70–94%). The mPFS was, respectively, 17 months (95% CI: 14 months to unreached) and unreached [14]. Among pre-treated patients, 55% had received anti-PD-1/PD-L1 antibodies. For pralsetinib, ORR was confirmed in 61% (95% CI 50–71%) of patients with previous platinum-based chemotherapy and in 70% (95% CI 50–86%) of treatment-naive patients [13]. Among previously treated patients, all patients had received chemotherapy, and 45% had received immunotherapy. Responses were observed regardless of previous anti-PD-1/PD-L1 therapy status. The median of PFS in pre-treated group of patients was 17.1 months (95% CI 8.3–22.1). Overall survival data for selective *RET*-inhibitors are still incomplete. Phase III studies comparing these drugs with chemotherapy or chemoimmunotherapy used in first-line treatment will provide more detailed data on survival parameters and safety profile. The safety of the treatment and possible interactions between TKIs and immunotherapy require special attention in the context of the sequential therapy.

Taking into account the data presented, which indicate questionable efficacy of immunotherapy and limited access to *RET*-inhibitors in the first-line treatment in patients with coexistent PD-L1 expression > 50% and *RET*-fusion, the use of chemotherapy based on platinum derivatives and pemetrexed may be considered.

### 4.2. Effectiveness of Immunotherapy in Patients with Other Rare Molecular Alterations

Should we expect the efficacy of immunotherapy in patients with simultaneous high PD-L1 expression and the presence of driver mutations in tumour tissue? Unquestionably, the effectiveness of immunotherapy has been proven in many clinical studies for PD-L1-expressed advanced NSCLC patients [28]. However, PD-L1 expression in tumours harbouring driver mutations does not necessarily correlate with response to single anti-PD-1 or anti-PD-L1 [4,5,18]. High expression of PD-L1 on tumour cells may reflect cell activation through PD-L1 pathway instead of being a marker of adaptive immune response. Moreover, different expressions of PD-L1 depending on the oncogenic driver mutation present in the tumour tissue have been observed in the lung cancer. *EGFR* mutation is usually connected with low PD-L1 expression, while *KRAS* and *MET* alterations are associated with higher PD-L1 tumour expression [4,5,18].

Mazieres et al. addressed the efficacy of ICIs in the context of oncogenic driver mutations. Due to a low number of patients with *ALK* (n = 23), *ROS1* (n = 7) and *RET* (n = 16) abnormalities in the entire group of 551 patients, these subgroups were analysed together as “rearrangements”. The ORR was 17% for *ROS1-*, 6% for *RET-* and 0% for *ALK*-bearing patients. In the entire cohort, mPFS was 2.8 months and OS—13.3 months. In the subgroup analysis, mPFS was 2.5 months for *ALK* and 2.1 months for *RET*-bearing patients [26]. The authors found out that ICIs induced regression in some tumours with actionable driver alterations, but overall clinical activity of immunotherapy was very low. They concluded that patients with actionable tumour alterations should receive targeted therapies and chemotherapy before considering single-agent immunotherapy [26]. The use of combination therapies, especially with multikinase inhibitors such as cabozantinib, lenvatinib, and vandetanib, could theoretically result in clinical response in these patients and requires prospective clinical trials in future.

A very interesting study on the effectiveness of immunotherapy in patients with *MET* gene alteration was conducted by Sabari et al. [29]. MET is a high affinity protooncogene receptor of tyrosine kinase that, upon activation, drives oncogenic pathways involved in cell proliferation, survival, and dissemination. MET inhibitors are active in patients with advanced *MET* exon 14-altered cancers and this abnormality is observed in 3–4% of NSCLC patients. Sabari et al. analysed the group of 147 *MET* exon 14-altered NSCLC patients at any stage in the terms of response to single-agent or combination ICIs [29]. The authors also assessed the PD-L1 molecule expression by immunochemistry staining and TMB by NGS panels. From this group, only 24 patients were evaluable for response—22 patients received single agent anti-PD-1/PD-L1 therapy, and two patients were given combination anti-PD-1 and anti-CTLA-4 therapy. The mPFS was 1.9 months (21 patients assessable for this end point), and the mOS was 18.2 months. The authors did not observe higher response rate in patients with either high PD-L1 expression (2/11, 18%) or in those with high TMB. To summarise, Sabari et al. concluded that the ORR with ICIs was low at 17% and was not distinctly different from efficacy in the unselected, second-line therapy [29].

A similarly designed study and its results was presented by Lai et al.; it evaluated the PD-L1 expression level and tumour mutation burden in *HER2*-mutant NSCLC patients with respect to their response to immunotherapy [30]. Within the group of 122 patients with *HER2*-alteration, 26 were treated with immunotherapy. ORR was 12%, including three patients with partial response, eight with stable disease and 15 with progressed disease [30]. Importantly, in the group of responders, none of the patients had *HER2*-alteration (2 patients expressed PD-L1 ≥ 50% and 2 patients had TMB ≥ median). From the immunotherapy starting time, mPFS was 1.9 months and mOS was 10.4 months. The authors concluded that those patients very rarely could benefit from immunotherapy. However, the authors believe that ICIs can still be considered in those patients, particularly in the context of high PD-L1 expression or higher TMB [30]. Similar data were presented by Neagro et al. [31]—three cohorts of patients with NSCLC were analysed (4189 patients in total) to assess the value of immunotherapy and the correlation between molecular alterations, PD-L1 expression and TMB [31]. PD-L1 expression > 50% was confirmed in about 20% of patients with *EGFR* mutations and in 34-55% of patients with other alterations (*ALK, BRAF V600E, ROS1, RET* and *MET*). According to multivariate analysis patients with *BRAF* V600E mutation (HR 0.58, *p* = 0.041), PD-L1 expression (HR 0.57, *p* < 0.01), and high TMB (HR 0.66, *p* < 0.001) benefited from the immunotherapy. Additionally, it was noted that the highest TMB (9.6 mut/Mb) had been observed in patients with *BRAF* non-V600E mutation (*p* < 0.001). In the group of patients with *BRAF* V600E mutations mTMB was <4 mut/Mb and the percentage of patients with high PD-L1 expression was high (45%). In the group of 118 patients with *BRAF* V600E mutation mPFS was 9.79 months and mOS was 20.8 months [31]. Lower median TMB (<3 mut/Mb) was observed in the patients with other alterations (*EGFR, ALK, RET, HER2,* and *ROS1*), which is a negative predictive factor for immunotherapy when accompanied by low PD-L1 expression. The authors also suggest other factors to be considered; those include low T cell receptor (TCR) clonality implying less reactive TCR, decreased proliferating and activated CD8+ T cell infiltration, lower IFN-ɤ expression, and increased TGF-ß signalling [31].

The study summarizing the efficacy of immunotherapy in patients with rare genetic abnormalities was presented by Enguren-Santamaria et al. [32]. The authors concluded that in general approach anti–PD-L1 monotherapy has a limited clinical impact in NSCLC patients with rare genetic alteration. The relevant immunotherapy clinical trials conducted exclusively in the druggable driver-positive NSCLC patients are unfortunately lacking now. The innovative therapeutic strategies, especially for this group of patients, are very much needed, including chemoimmunotherapy strategies, synergistic immunotherapy combinations, and adoptive cell therapies. Simultaneously, deep knowledge about the tumour biology and the specific immune escape mechanisms could help make a right decision [32].

## 5. Conclusions

Despite the high PD-L1 expression, no benefit was obtained from the use of pembrolizumab in our *RET*-fused patients. Based on the data from the literature, single-agent immunotherapy seems to be ineffective in this group of patients, just like in patients with other rare molecular alterations. Combined chemoimmunotherapy can result in improved effectiveness. Targeted drugs, if available, are the optimal treatment for patients with rare molecular alterations, including *RET* gene related, but the optimal sequence of targeted therapy, chemotherapy, and immunotherapy is not defined. *RET*-inhibitors are effective regardless of previous treatment (including platinum-based chemotherapy with or without immunotherapy and untreated patients); the incidence of treatment-related hypersensitivity reactions may be higher in patients after immunotherapy [33].

## Figures and Tables

**Table 1:** Clinical outcomes with immune-checkpoint inhibitors monotherapy in *RET*-rearranged lung cancer patients.

Author	Number of Patients	Number of Patients with PD-L1 > 50%	Response	Efficacy
Rodriguez 2021 [23]	6	3	6 *	mPFS 9 months
Baglivo 2020 [24]	2	2	PD in both patients	PD after 1 cycle of pembrolizumab in both patients
Riudavets 2020 [25]	1	1	CR	Treatment discontinuation due to toxicity
Offin 2019 [22]	16	1	Assessed in 13 patients: PD-8 (62%), SD-3 (23%), non-CR/non-PD—2 (15%)	mPFS 3.4 months (95% CI 2.1–5.6) Patient with PD-L1 > 50%: PD after 1.3 month (nivolumab plus ipilimumab)
Mazieres 2019 [26]	16	NA	PR-1, SD-3 (25%) PD-12 (75%)	mPFS 2.1 months (95% CI 1.3–4.7) mOS 21.3 months (95% CI 3.8–28)
Dudnik 2018 [5]	13	1	Assessed in 4 patients- ORR 0/4	mPFS 3.0 months (95% CI 1.9–3.1) mOS 14.9 months (95% CI 7.2–19.7)
Guisier 2019 [18]	9	2	PR-3, SD-2, PD-3, NA-1	mPFS 7.6 months (95% CI 2.3-NR) mOS NR, 12 months OS 88.9% pts
Lee 2020 [21]	13	NA	ORR-0% DCR-25–50%	mPFS 2.1 months (95% CI: 1.6–2.6) mOS 12.4 (95% CI: 2.9–21.8)
Bhandari 2021 [22]	69	NA	** ORR-33% DCR 66%	mPFS 4,2 months (95% CI: 1.4–8.4) mOS 19.1 months (95% CI: 6.9–NR)
Baby 2021 [27]	1	1	CR	tumour response lasting for 29 months and ongoing

Abbreviations: PD—progressive disease; CR—complete response; SD—stable disease; PR—partial response, mPFS—median progression free survival, mOS—median overall survival, *—no detailed data, **—11 patients in the second line setting, monotherapy.

## References

[1] Reck M., Rodríguez-Abreu D., Robinson A.G., Hui R., Csőszi T., Fülöp A., Gottfried M., Peled N., Tafreshi A., Cuffe S. (2016). Pembrolizumab versus Chemotherapy for PD-L1–Positive Non–Small-Cell Lung Cancer. N. Engl. J. Med..

[2] Reck M., Rodríguez-Abreu D., Robinson A.G., Hui R., Csőszi T., Fülöp A., Gottfried M., Peled N., Tafreshi A., Cuffe S. (2021). Five-Year Outcomes With Pembrolizumab Versus Chemotherapy for Metastatic Non–Small-Cell Lung Cancer With PD-L1 Tumor Proportion Score ≥ 50. J. Clin. Oncol..

[3] Rittmeyer A., Barlesi F., Waterkamp D., Park K., Ciardiello F., von Pawel J., Gadgeel S.M., Hida T., Kowalski D.M., Dols M.C. (2017). Atezolizumab versus docetaxel in patients with previously treated non-small-cell lung cancer (OAK): A phase 3, open-label, multicentre randomised controlled trial. Lancet.

[4] Calles A., Riess J.W., Brahmer J.R. (2020). Checkpoint Blockade in Lung Cancer With Driver Mutation: Choose the Road Wisely. Am. Soc. Clin. Oncol. Educ. Book.

[5] Dudnik E., Bshara E., Grubstein A., Fridel L., Shochat T., Roisman L.C., Ilouze M., Rozenblum A.B., Geva S., Zer A. (2018). Rare targetable drivers (RTDs) in non-small cell lung cancer (NSCLC): Outcomes with immune check-point inhibitors (ICPi). Lung Cancer.

[6] Gautschi O., Milia J., Filleron T., Wolf J., Carbone D.P., Owen D.H., Camidge R., Narayanan V., Doebele R.C., Besse B. (2017). Targeting RET in Patients With RET-Rearranged Lung Cancers: Results From the Global, Multicenter RET Registry. J. Clin. Oncol..

[7] Hess L.M., Han Y., Zhu Y.E., Bhandari N.R., Sireci A. (2021). Characteristics and outcomes of patients with RET-fusion positive non-small lung cancer in real-world practice in the United States. BMC Cancer.

[8] Drilon A., Bergagnini I., Delasos L., Sabari J., Woo K.M., Plodkowski A., Wang L., Hellmann M.D., Joubert P., Sima C.S. (2016). Clinical outcomes with pemetrexed-based systemic therapies in RET-rearranged lung cancers. Ann. Oncol..

[9] Shen T., Pu X., Wang L., Yu Z., Li J., Zhang Y., Liang X., Chen H., Xu C., Song Z. (2020). Association Between RET Fusions and Efficacy of Pemetrexed-based Chemotherapy for Patients With Advanced NSCLC in China: A Multicenter Retrospective Study. Clin. Lung Cancer.

[10] Hida T., Velcheti V., Reckamp K.L., Nokihara H., Sachdev P., Kubota T., Nakada T., Dutcus C.E., Ren M., Tamura T. (2019). A phase 2 study of lenvatinib in patients with RET Fusion-positive lung adenocarcinoma. Lung Cancer.

[11] Drilon A., Rekhtman N., Arcila M., Wang L., Ni A., Albano M., van Voorthuysen M., Somwar R., Smith R.S., Montecalvo J. (2016). Cabozantinib in patients with advanced RET -rearranged non-small-cell lung cancer: An open-label, single-centre, phase 2, single-arm trial. Lancet Oncol..

[12] Yoh K., Seto T., Satouchi M., Nishio M., Yamamoto N., Murakami H., Nogami N., Nosaki K., Kohno T., Tsuta K. (2021). Vantedanib in patients with previously treated RET-rearranged advanced non-small-cell lung cancer (LURET): An open-label, multicentre phase 2 trial. Lung Cancer.

[13] Gainor J.F., Curigliano G., Kim D.-W., Lee D.H., Besse B., Baik C.S., Doebele R.C., Cassier P.A., Lopes G., Tan D.S.W. (2021). Pralsetinib for RET fusion-positive non-small-cell lung cancer (ARROW): A multi-cohort, open-label, phase 1/2 study. Lancet Oncol..

[14] Drilon A., Oxnard G.R., Tan D.S., Loong H.H., Johnson M., Gainor J., McCoach C.E., Gautschi O., Besse B., Cho B.C. (2020). Efficacy of Selpercatinib in RET Fusion-Positive Non-Small-Cell Lung Cancer. N. Engl. J. Med..

[15] Offin M., Guo R., Wu S.L., Sabari J., Land J.D., Ni A., Montecalvo J., Halpenny D.F., Buie L.W., Pak T. (2019). Immunophenotype and Response to Immunotherapy of RET-Rearranged Lung Cancers. JCO Precis. Oncol..

[16] Dantoing E., Piton N., Salaün M., Thiberville L., Guisier F. (2021). Anti-PD1/PD-L1 Immunotherapy for Non-Small Cell Lung Cancer with Actionable Oncogenic Driver Mutations. Int. J. Mol. Sci..

[17] Greillier L., Tomasini P., Barlesi F. (2018). The clinical utility of tumor mutational burden in non-small cell lung cancer. Transl. Lung Cancer Res..

[18] Schoenfeld A.J., Rizvi H., Bandlamudi C., Sauter J.L., Travis W.D., Rekhtman N., Plodkowski A.J., Perez-Johnston R., Sawan P., Beras A. (2020). Clinical and molecular correlates of PD-L1 expression in patients with lung adenocarcinomas. Ann. Oncol..

[19] Guisier F., Dubos-Arvis C., Viñas F., Doubre H., Ricordel C., Ropert S., Janicot H., Bernardi M., Fournel P., Lamy R. (2020). Efficacy and safety of anti-PD-1 immunotherapy in patients with advanced non-small cell lung cancer with BRAF, HER2 or MET mutation or RET-translocation: GFPC 01-2018. J. Thorac. Oncol..

[20] Hegde A., Huang L., Liu S., Hess K., Cabanillas M., Hu M., Busaidy N., Sherman S., Simon G., Blumenschein G. (2019). Abstract 4997: Responsiveness to immune checkpoint inhibitors in RET dependent cancers. Cancer Res..

[21] Lee J., Ku B.M., Shim J.H., La Choi Y., Sun J.-M., Lee S.-H., Ahn J.S., Park K., Ahn M.-J. (2020). Characteristics and outcomes of RET-rearranged Korean non-small cell lung cancer patients in real-world practice. Jpn. J. Clin. Oncol..

[22] Bhandari N.R., Hess L.M., Han Y., Zhu Y.E., Sireci A.N. (2021). Efficacy of immune checkpoint inhibitor therapy in patients with RET fusion-positive non-small-cell lung cancer. Immunotherapy.

[23] Rodriguez E., Dawar R., Gawri K., Thammineni V., Torres T., Fanfan D., Saul E., Ikpeazu C., Lopes G. (2021). Chemotherapy and immunotherapy outcomes of RET-rearranged lung cancers: A Case Series. J. Thorac. Oncol..

[24] Baglivo S., Ludovini V., Moretti R., Bellezza G., Sidoni A., Roila F., Metro G. (2020). RET Rearrangement as a Predictor of Unresponsiveness to Immunotherapy in Non-Small Cell Lung Cancer: Report of Two Cases with Review of the Literature. Oncol. Ther..

[25] Riudavets M., Caramella C., Pradere P., Naltet C., Le Pechoux C., Adam J., Mabille L., Rouleau E., Besse B., Planchard D. (2020). Complete, unpredictable, multi-site response including brain and liver metastases in a patient with RET-rearranged non-small-cell Lung cancer treated with single-agent immunotherapy: A case report. Clin. Lung Cancer.

[26] Mazieres J., Drilon A., Lusque A.B., Mhanna L., Cortot A., Mezquita L., Thai A.A., Mascaux C., Couraud S., Veillon R. (2019). Immune checkpoint inhibitors for patients with advanced lung cancer and oncogenic driver alterations: Results from the IMMUNOTARGET registry. Ann. Oncol..

[27] Baby S., Khalil F., Tanvetyanon T. (2021). Frontline pembrolizumab for the treatment of RET-rearranged non-small cell lung cancer: A case report. Cancer Treat. Res. Commun..

[28] Bodor J.N., Boumber Y., Borghaei H. (2020). Biomarkers for immune checkpoint inhibition in non-small cell lung cancer (NSCLC). Cancer.

[29] Sabari J., Leonardi G., Shu C., Umeton R., Montecalvo J., Ni A., Chen R., Dienstag J., Mrad C., Bergagnini I. (2018). PD-L1 expression, tumor mutational burden, and response to immunotherapy in patients with MET exon 14 altered lung cancers. Ann. Oncol..

[30] Lai W.C.V., Feldman D.L., Buonocore D.J., Brzostowski E.B., Rizvi H., Plodkowski A.J., Ni A., Sabari J.K., Offin M.D., Kris M.G. (2018). PD-L1 expression, tumor mutation burden and response to immune checkpoint blockade in patients with HER2-mutant lung cancers. J. Clin. Oncol..

[31] Negrao M.V., Skoulidis F., Montesion M., Schulze K., Bara I., Shen V., Xu H., Hu S., Sui D., Elamin Y.Y. (2021). Oncogene-specific differences in tumor mutational burden, PD-L1 expression, and outcomes from immunotherapy in non-small cell lung cancer. J. Immunother. Cancer.

[32] Eguren-Santamaria I., Sanmamed M.F., Gil-Bazo I. (2020). Are Immune Checkpoint Inhibitors Effective Against Uncommon Oncogene-Driven NSCLC Subtypes?. J. Thorac. Oncol..

[33] McCoach C.E., Rolfo C., Drilon A., Lacouture M., Besse B., Goto K., Zhu V.W., Tan D.S., Farajian S., Potter L.A. (2022). Hypersensitivity Reactions to Selpercatinib Treatment with or Without Prior Immune Checkpoint Inhibitor Therapy in Patients with Non-Small-Cell Lung Cancer in LIBRETTO-001. J. Thorac. Oncol..

